# Annual Commencement

**Published:** 1859-02

**Authors:** 


### EDITORIAL.

### ANNUAL COMMENCEMENT.

The Sixteenth Annual Commencement of Rush Medical College, took place on Wednesday afternoon, the 16th of February, in the hall of the College. The hall was well filled with an intelligent audience, and the exercises were varied and interesting.

After a prayer by the Rev. Mr. Schenck, Mr. Benj. W. Bristow, one of the candidates for graduation, read his Thesis on the Qualifications of the Physician. The degree of Doctor of Medicine was then conferred on thirty-one members of the class. Three awards, each consisting of a valuable medical work, were then distributed as follows: 1st, to Wm. E. Peters, for having sustained the best examination in the several departments. 2d, to John W. W. Lawrence, for the best Inaugural Thesis. 3d, to L. G. Armstrong, for the best examination in surgery. An able and eloquent Valedictory Address was then delivered by Prof. D. Brainard; and the public exercises were closed with a benediction.

After the audience had retired, the graduates and members of the college class, with the faculty and invited guests, repaired to an adjoining room, and spent a couple of hours around the festive board, in the highest degree of social and intellectual enjoyment.

The following are the names of the Graduates, viz:

| | |
|---|---|
| Wm. L. Kreider, | J. F. Williams, |
| John A. Cook, | Blexton Harris, |
| L. G. Armstrong, | J. R. Pearce, |
| Richard Hull, | E. H. Ayres, |
| Geo. W. Corey, | John H. Frizell, |
| John W. W. Lawrence, | N. M. Douthett, |
| E. Livingston Welling, | W. H. Lyford, |
| Myron Underwood, | Lafayette Lake, |
| Richard McGee, | A. M. Blackman, |
| R. F. Williams, | Wm. E. Peters, |
| Benj. W. Bristow, | F. Mason, |
| A. B. Taylor, | P. R. Slingsby, |
| J. H. Wiley, | Sam'l McNair, |
| J. R. Conklin, | E. C. Dickinson, |
| E. A. Steele, | E. O. F. Roler. |
| Wm. C. Hopwood, | |

The honorary degree of M.D. was conferred on Dr. S. M. Mitchell, of Illinois; and the *ad-cundem* degree on J. Drake Harpur, of Springfield, Ill.

